# A Formal Treatment of Sequential Ignorability

**DOI:** 10.1007/s12561-014-9110-8

**Published:** 2014-04-08

**Authors:** A. Philip Dawid, Panayiota Constantinou

**Affiliations:** 1 Statistical Laboratory, Centre for Mathematical Sciences, University of Cambridge, Wilberforce Road, Cambridge , CB3 0WB UK; 2 Department of Mathematics, University of Bristol, University Walk, Clifton, Bristol , BS8 1TW UK

**Keywords:** Causal inference, $G$-computation, Influence diagram, Observational study, Sequential decision theory, Stability

## Abstract

Taking a rigorous formal approach, we consider sequential decision problems involving observable variables, unobservable variables, and action variables. We can typically assume the property of *extended stability*, which allows identification (by means of “$G$-computation”) of the consequence of a specified treatment strategy if the “unobserved” variables are, in fact, observed—but not generally otherwise. However, under certain additional special conditions we can infer *simple stability* (or *sequential ignorability*), which supports $G$-computation based on the observed variables alone. One such additional condition is *sequential randomization*, where the unobserved variables essentially behave as random noise in their effects on the actions. Another is *sequential irrelevance*, where the unobserved variables do not influence future observed variables. In the latter case, to deduce sequential ignorability in full generality requires additional positivity conditions. We show here that these positivity conditions are not required when all variables are discrete.

## Introduction

We are often concerned with controlling some variable of interest through a sequence of consecutive actions. An example in a medical context is maintaining a critical variable, such as blood pressure, within an appropriate risk-free range. To achieve such control, the doctor will administer treatments over a number of stages, taking into account, at each stage, a record of the patient’s history, which provides him with information on the level of the critical variable, and possibly other related measurements, as well as the patient’s reactions to the treatments applied in preceding stages. Consider, for instance, practices followed after events such as stroke, pulmonary embolism or deep vein thrombosis [18, 19]. The aim of such practices is to keep the patient’s prothrombin time (international normalized ratio, INR) within a recommended range. Such efforts are not confined to a single decision and instant allocation of treatment, marking the end of medical care. Rather, they are effected over a period of time, with actions being decided and applied at various stages within this period, based on information available at each stage. So the patient’s INR and related factors will be recorded throughout this period, along with previous actions taken, and at each stage all the information so far recorded, as well, possibly, as other, unrecorded information, will form the basis upon which the doctor will decide on allocation of the subsequent treatment.

A well-specified algorithm that takes as input the recorded history of a patient at each stage and gives as output the choice of the next treatment to be allocated constitutes a *dynamic decision strategy*. Such a strategy gives guidance to the doctor on how to take into account the earlier history of the patient, including reactions to previous treatments, in allocating the next treatment. There can be an enormous number of such strategies, having differing impacts on the variable of interest. We should like to have criteria to evaluate these strategies, and so allow us to choose the one that is optimal for our problem [11].

In this paper we develop and extend the decision-theoretic approach to this problem described by Dawid and Didelez [9]. A problem that complicates the evaluation of a strategy is that the data we possess were typically not generated by applying that strategy, but arose instead from an observational study. We thus seek conditions, which we shall express in decision-theoretic terms, under which we can identify the components we need to evaluate a strategy from such data. When appropriate conditions are satisfied, the *G-computation algorithm* introduced by Robins [13, 16] allows us to evaluate a strategy on the basis of observational data. Our decision-theoretic formulation of this is closely related to the seminal work of Robins [13–15, 17], but is, we consider, more readily interpretable.

The plan of the paper is as follows. In Sect. 2 we detail our notation, and describe the *G-recursion algorithm* for evaluating an interventional strategy. We next discuss the problem of identifiability, which asks when observational data can be used to evaluate a strategy. Distinguishing between the observational and interventional regimes, we highlight the need for conditions that would allow us to transfer information across regimes, and thus support observational evaluation of an interventional strategy.

In Sect. 3 we describe the decision-theoretic framework by means of which we can formulate such conditions formally in a simple and comprehensible way, and so address our questions. In particular, we show how the language and calculus of conditional independence supply helpful tools that we can exploit to attack the problem of evaluating a strategy from observational data.

In Sect. 4 we introduce *simple stability*, the most straightforward condition allowing us to evaluate a strategy, by means of $G$-recursion, from observational data. However, in many problems this condition is not easily defensible, so in Sect. 5 we explore other conditions: in particular, conditions we term *sequential randomization* and *sequential irrelevance*. We investigate when these are sufficient to induce simple stability (and therefore observational evaluation of a strategy), and discuss their limitations. In particular, we show that, when all variables are discrete, we can drop the requirement of positivity that is otherwise required to deduce simple stability when sequential irrelevance holds. Counter-example 5.5, as well as Counter-example A.1 and A.2 in the Appendix, shows the need for positivity in more general problems. Section 7 presents some concluding comments.

## A Sequential Decision Problem

We are concerned with evaluating a specified multistage procedure that aims to affect a specific outcome variable of interest through a sequence of interventions, each responsive to observations made thus far. As an example we can take the case of HIV disease. We consider evaluating strategies that, aiming to suppress the virus and stop disease progression, recommend when to initiate antiretroviral therapy for HIV patients based on their history record. This history will take into account the CD4 count [19], as well as additional variables relevant to the disease.

### Notation and Terminology

We consider two sets of variables: $\mathcal {L}$, a set of *observable* variables, and $\mathcal {A}$, a set of *action* variables. We term the variables in $\mathcal {L} \cup \mathcal {A}$ *domain variables*. An alternating ordered sequence $\mathcal{I}:= (L_1, A_1,\ldots , L_n, A_n, L_{n+1}\equiv Y)$ with $L_i\subseteq \mathcal{L}$ and $A_i\in \mathcal{A}$ defines an *information base*, the interpretation being that the specified variables are observed in this time order. We shall adopt notational conventions such as $(L_1, L_2)$ for $L_1 \cup L_2$, $\overline{L}_i$ for $(L_1, \ldots ,L_i)$, etc.

The observable variables $\mathcal{L}$ represent initial or intermediate symptoms, reactions, personal information, etc., observable between consecutive treatments, over which we have no direct control; they are perceived as generated and revealed by Nature. The action variables $\mathcal{A}$ represent the treatments, which we could either control by external intervention, or else leave to Nature to determine. Thus at each stage $i$ we shall have a realization of the random variable or set of random variables $L_i \subseteq \mathcal {L}$, followed by a value for the variable $A_i \in \mathcal {A}$. After the realization of the final $A_n \in \mathcal {A}$, we observe the outcome variable $L_{n+1} \in \mathcal {L}$, which we also denote by $Y$.

A configuration $h_i :=(l_1, a_1, \ldots , a_{i-1}, l_i)$ of the variables $(L_1, A_1,\ldots , A_{i-1}, L_i)$, for any stage $i$, constitutes a *partial history*. A clearly described way of specifying, for each action $A_i$, its value $a_i$ as a function of the partial history $h_i$ to date defines a *strategy*: the values $(\overline{l}_i, \overline{a}_{i-1})$ of the earlier domain variables $(\overline{L}_i, \overline{A}_{i-1})$ can thus be taken into account in determining the current and subsequent actions.

In a *static*, or *atomic*, strategy, the sequence of actions is predetermined, entirely unaffected by the information provided by the $L_i$’s. In a *non-randomized dynamic strategy* we specify, for each stage $i$ and each partial history $h_i$, a fixed value $a_i$ of $A_i$, that is then to be applied. We can also consider *randomized strategies*, where for each stage $i$ and associated partial history $h_i$ we specify a probability distribution for $A_i$, so allowing randomization of the decision for the next action. In this paper we consider general randomized strategies, since we can regard static and non-randomized strategies as special cases of these. Then all the $L_i$’s and $A_i$’s have the formal status of random variables. We write e.g. $\mathbb {E}(L_i \mid \overline{A}_{i-1},\overline{L}_{i-1}\,;\, s)$ to denote any version of the conditional expectation $\mathbb {E}(L_i \mid \overline{A}_{i-1},\overline{L}_{i-1})$ under the joint distribution $P_{s}$ generated by following strategy $s$, and “$\hbox {a.s.}\;{P_s}$” to denote that an event has probability 1 under $P_{s}$.

### Evaluating a Strategy

Suppose we want to identify the effect of some strategy $s$ on the outcome variable $Y$: we then need to be able to assess the overall effect that the action variables have on the distribution of $Y$. An important application is where we have a loss $L(y)$ associated with each outcome $y$ of $Y$, and want to compute the expected loss $\mathbb {E}\{L(Y)\}$ under the distribution for $Y$ induced by following strategy $s$. We shall see in Sect. 4 below that, if we know or can estimate the conditional distribution, under this strategy, of each observable variable $L_i$ ($i=1,\ldots ,n+1$) given the preceding variables in the information base, then we would be able to compute $\mathbb {E}\{L(Y)\}$. Following this procedure for each contemplated strategy, we could compare the various strategies, and so choose that minimizing expected loss.

In order to evaluate a particular strategy of interest, we need to be able to mimic the experimental settings that would give us the data we need to estimate the probabilistic structure of the domain variables. Thus suppose that we wish to evaluate a specified non-randomized strategy for a certain patient $P$, and consider obtaining data under two different scenarios.

The first scenario corresponds to precisely the strategy that we wish to evaluate: that is, the doctor knows the prespecified plan defined by the strategy, and at each stage $i$, taking into account the partial history $h_i$, he allocates to patient $P$ the treatment that the strategy recommends. The expected loss $\mathbb {E}\{L(Y)\}$ computed under the distribution of $Y$ generated by following this strategy is exactly what we need to evaluate it.

Now consider a second scenario. Patient $P$ does not take part in the experiment described above, but it so happens he has received exactly the same sequence of treatments that would be prescribed by that strategy. However, the doctor did not decide on the treatments using the strategy, but based on a combination of criteria, that might have involved variables beyond the domain variables $\mathcal {L} \cup \mathcal {A}$. For example, the doctor might have taken into account, at each stage, possible allergies or personal preferences for certain treatments of patient $P$, variables that the strategy did not encompass.

Because these extra variables are not recorded in the data, the analyst does not know them. Superficially, both scenarios appear to be the same, since the variables recorded in each scenario are the same. However, without further assumptions there is no reason to believe that they have arisen from the same distribution.

We call the regime described in the first scenario above an *interventional regime*, to reflect the fact that the doctor was intervening in a specified fashion (which we assume known to the analyst), according to a given strategy for allocating treatment. We call the regime described in the second scenario an *observational regime*, reflecting the fact that the analyst has just been observing the sequence of domain variables, but does not know just how the doctor has been allocating treatments.

Data actually generated under the interventional regime would provide exactly the information required to evaluate the strategy. However, typically the data available will not have been generated this way—and in any case there are so many possible strategies to consider that it would not be humanly possible to obtain such experimental data for all of them. Instead, the analyst may have observed how patients (and doctors) respond, in a single, purely observational, regime. Direct use of such observational data, as if generated by intervention, though tempting, can be very misleading. For example, suppose the analyst wants to estimate, at each stage $i$, the conditional distribution of $L_i$ given $(\overline{L}_{i-1}, \overline{A}_{i-1})$ in the interventional regime (which he has not observed), using data from the observational regime (which he has). Since all the variables in this conditional distribution have been recorded in the observational regime, he might instead estimate (as he can) the conditional distribution of $L_i$ given $(\overline{L}_{i-1}, \overline{A}_{i-1})$ in the observational regime, and consider this as a proxy for its interventional counterpart. However, since the doctor may have been taking account of other variables, which the analyst has not recorded and so can not adjust for, this estimate will typically be biased, often seriously so. One of the main aims of this paper is to consider conditions under which the bias due to such potential confounding disappears.

For simplicity, we assume that all the domain variables under consideration can be observed for every patient. However, the context in which we observe these variables will determine if and how we can use the information we collect. The decision-theoretic approach we describe below takes into account the different circumstances of the different regimes by introducing a parameter to identify which regime is under consideration at any point. In order to tackle issues such as the potential for bias introduced by making computations under a regime distinct from that we are interested in evaluating, we need to make assumptions relating the probabilistic behaviours under the differing regimes. Armed with such understanding of the way the regimes interconnect, we can then investigate whether, and if so how, we can transfer information from one regime to another.

### Consequence of a Strategy

We seek to calculate the expectation $\mathbb {E}\{k(Y)\,;\, {s}\}$ (always assumed to exist) of some given function $k(\cdot )$ of $Y$ in a particular interventional regime $s$; for example, $k(\cdot )$ could be a loss function, $k(y) \equiv L(y)$, associated with the outcome $y$ of $Y$. We shall use the term *consequence* of $s$ to denote the expectation $\mathbb {E}\{k(Y)\,;\, {s}\}$ of $k(Y)$ under the contemplated interventional regime $s$.

Assuming $(L_1,A_1, \ldots , L_N, A_N, Y)$ has a joint density in interventional regime $s$, we can factorize it as:

1 $$\begin{aligned} p(y, \overline{l}, \overline{a}\,;\, {s}) = \left\{ \prod _{i=1}^{n+1} p(l_i \mid \overline{l}_{i-1}, \overline{a}_{i-1}\,;\, {s})\right\} \times \left\{ \prod _{i=1}^n p(a_i \mid \overline{l}_{i}, \overline{a}_{i-1}\,;\, {s})\right\} \end{aligned}$$

with $$l_{n+1} \equiv y$$.

#### $G$-recursion

If we knew all the terms on the right-hand side of (1), we could in principle compute the joint density for $(Y, \overline{L}, \overline{A})$ under strategy $s$, hence, by marginalization, the density of $Y$, and finally the desired consequence $\mathbb {E}\{k(Y);s\}$. However, a more efficient way to compute this is by means of the *G-computation* formula introduced by Robins [13]. Here we describe the recursive formulation of this formula, *G-recursion*, as presented in Dawid and Didelez [9].

Let $h$ denote a partial history of the form $(\overline{l}_i, \overline{a}_{i-1})$ or $(\overline{l}_i, \overline{a}_i)$ ($0 \le i \le n+1)$. We denote the set of all partial histories by $\mathcal{H}$. Fixing a regime $s \in \mathcal{S}$, define a function $f$ on $\mathcal{H}$ by:

2 $$\begin{aligned} f(h) := \mathbb {E}\{k(Y) \mid h\,;\, s\}. \end{aligned}$$

##### Note:

When we are dealing with non-discrete distributions (and also in the discrete case when there are non-trivial events of $P_s$-probability 0), the conditional expectation on the right-hand side of (2) will not be uniquely defined, but can be altered on a set of histories that has $P_s$-probability 0. Thus we are in fact requiring, for each $i$:

3 $$\begin{aligned} f(\overline{L}_i, \overline{A}_{i}) := \mathbb {E}\{k(Y) \mid \,\overline{L}_i, \overline{A}_{i};\, s\}\quad \mathrm{{a.s.}}\; [{P_s}] \end{aligned}$$

(and similarly when the argument is $(\overline{L}_i, \overline{A}_{i-1})$). And we allow the left-hand side of (2) to denote *any* selected version of the conditional expectation on the right-hand side.

For any versions of these conditional expectations, applying the law of repeated expectation yields:

4 $$\begin{aligned} f(\overline{L}_i, \overline{A}_{i-1})&= \mathbb {E}\left\{ f(\overline{L}_i, \overline{A}_i)\, \mid \, \overline{L}_{i}, \overline{A}_{i-1}\,;\, s)\right\} \quad \hbox {a.s.}\;[{P_s}]\end{aligned}$$

5 $$\begin{aligned} f(\overline{L}_{i-1}, \overline{A}_{i-1})&= \mathbb {E}\left\{ f(\overline{L}_i, \overline{A}_{i-1}\, \mid \, \overline{L}_{i-1}, \overline{A}_{i-1}\,;\,s)\right\} \hbox {a.s.}\;[{P_s}]. \end{aligned}$$

For $h$ a full history $(\overline{l}_n, \overline{a}_n, y)$, we have $f(h) = k(y)$. Using these starting values, by successively implementing (4) and (5) in turn, starting with (5) for $i = n+1$ and ending with (5) for $i=1$, we step down through ever shorter histories until we have computed $f(\emptyset ) = \mathbb {E}\{k(Y)\,;\,s\}$, the consequence of regime $s$. Note that this equality is only guaranteed to hold almost surely, but since both sides are constants they must be the same constant. In particular, it can not matter which version of the conditional expectations we have chosen in conducting the above recursion: in all cases we will exit with the desired consequence $\mathbb {E}\{k(Y)\,;\,s\}$.

### Using Observational Data

In order to compute $\mathbb {E}\{k(Y)\,;\,s\}$, whether directly from (1) or using $G$-recursion, (4) and (5), we need (versions of) the following conditional distributions under $P_s$:

(i) $$A_i \,\mid \, \overline{L}_{i}, \overline{A}_{i-1}$$, for $$i = 1, \ldots ,n$$.
(ii) $$L_i \,\mid \, \overline{L}_{i-1}, \overline{A}_{i-1}$$, for $$i = 1, \ldots , n+1$$.

Since $s$ is an interventional regime, corresponding to a well-defined (possibly randomized) treatment strategy, the conditional distributions in (i) are fully specified by the treatment protocol. So we only need to get a handle on each term of the form (ii). However, since we have not implemented the strategy $s$, we do not have data directly relevant to this task. Instead, we have observational data, arising from a joint distribution we shall denote by $P_o$. We might then be tempted to replace the desired but not directly accessible conditional distribution, under $P_s$, of $L_i\, \mid \overline{L}_{i-1}, \overline{A}_{i-1}$, by its observational counterpart, computed under $P_o$, which is (in principle) estimable from observational data. This will generally be a dangerous ploy, since we are dealing with two quite distinct regimes, with strong possibilities for confounding and other biases in the observational regime; however, it can be justifiable if we can impose suitable extra conditions, relating the probabilistic behaviours of the different regimes. We therefore now turn to a description of a general “decision-theoretic” framework that is useful for expressing and manipulating such conditions.

## The Decision-Theoretic Approach

In the decision-theoretic approach to causal inference, we proceed by making suitable assumptions relating the probabilistic behaviours of stochastic variables across a variety of different regimes. These could relate to different locations, time-periods, or, in this paper, contexts (observational/interventional regimes) in which observations can be made. We denote the set of all regimes under consideration by $\mathcal{S}$. We introduce a non-stochastic variable $\sigma$, the *regime indicator*, taking values in $\mathcal{S}$, to index these regimes and their associated probability distributions. Thus $\sigma$ has the logical status of a parameter, rather than a random variable: it specifies which (known or unknown) joint distribution is operating over the domain variables $\mathcal{L}\cup \mathcal{A}$. Any probabilistic statement about the domain variables must, explicitly or implicitly, be conditional on some specified value $s\in \mathcal{S}$ for $\sigma$.

We focus here on the case that we want to make inference about one or more interventional regimes on the basis of data generated under an observational regime. So we take $\mathcal{S}=\{o\} \cup \mathcal{S}^*$, where $o$ is the observational regime under which data have been gathered, and $\mathcal{S}^*$ is the collection of contemplated interventional strategies with respect to a given information base $(L_1, A_1,\ldots , L_N, A_N, Y)$.

### Conditional Independence

In order to address the problem of making inference from observational data we need to assume (and justify) some relationships between the probabilistic behaviours of the variables in the differing regimes, interventional and observational. These assumptions will typically relate certain conditional distributions across different regimes. The notation and calculus of *conditional independence* (CI) turn out to be well-suited to express and manipulate such assumptions.

#### Conditional Independence for Stochastic Variables

Let $X, Y, Z, \ldots$ be random variables defined on the same probability space $(\varOmega ,\mathcal{A},P)$. We write $X \, \,\perp \!\!\!\perp \, Y \mid Z\,\,\, [P]$, or just $X \, \,\perp \!\!\!\perp \, Y \mid Z$ when $P$ is understood, to denote that *X is independent of Y given Z* under $P$: this can be interpreted as requiring that the conditional distribution, under $P$, of $X$, given $Y=y$ and $Z=z$, depends only on $y$ and not further on the value $z$ of $Z$. More formally, we require that, for any bounded real measurable function $h(X)$, there exists a measurable function $w(Z)$ such that

6 $$\begin{aligned} \mathbb {E}\{h(X) \,|\,Y, Z\} = w(Z)\quad \hbox {a.s.}\;[{P}]. \end{aligned}$$

Stochastic CI so defined has various general properties, of which the most important are the following—which can indeed be used as axioms of an independent “calculus of CI” [3, 7, 12].

##### **Theorem 3.1**

1. (Symmetry) $$X \, \,\perp \!\!\!\perp \, Y \mid Z \Rightarrow Y \, \,\perp \!\!\!\perp \, X \mid Z$$
2. $$X \, \,\perp \!\!\!\perp \, Y \mid X$$
3. (Decomposition) $$X \, \,\perp \!\!\!\perp \, Y \mid Z$$ and $$W \preceq Y$$ $$\Rightarrow $$ $$X \, \,\perp \!\!\!\perp \, W \mid Z$$
4. (Weak Union) $X \, \,\perp \!\!\!\perp \, Y \mid Z$ and $W \preceq Y$ $\Rightarrow X \, \,\perp \!\!\!\perp \, Y \mid (W,Z)$
5. (Contraction) $$X \, \,\perp \!\!\!\perp \, Y \mid Z$$ and $$X \, \,\perp \!\!\!\perp \, W \mid (Y,Z)$$ $$\Rightarrow X \, \,\perp \!\!\!\perp \, (Y,W) \mid Z$$

(Here $W \preceq Y$ is used to denote that $W =f(Y)$ for some measurable function $f$). These properties can be shown to hold universally for random variables on a common probability space [1] [Theorem 3.2.29].

#### Extended Conditional Independence

We can generalize the property $X \, \,\perp \!\!\!\perp \, Y \mid Z$ by allowing either or both of $Y, Z$ to be or contain non-stochastic elements, such as parameters or regime indicators [3, 5, 6]: in this case we talk of *extended conditional independence*. Thus let $\sigma$ denote the non-stochastic regime indicator. Informally, we interpret $X \, \,\perp \!\!\!\perp \, \sigma \mid Z$ as saying that the conditional distribution of $X$, given $Z=z$, under regime $\sigma =s$, depends only on $z$ and not further on the value $s$ of $\sigma$; that is to say, the conditional distribution of $X$ given $Z$ is the same in all regimes. Note that this is exactly the form of “causal assumption”, allowing transfer of probabilistic information across regimes, that we might wish to apply.

More formally, let $\{P_s: s \in \mathcal{S}\}$ be a family of distributions, and $X, Y, Z$,...random variables, on a measure space $(\varOmega , \mathcal{A})$. We introduce the non-stochastic regime indicator variable $\sigma$ taking values in $\mathcal{S}$, and interpret conditioning on $\sigma =s$ to mean that we are computing under distribution $P_s$.

##### **Definition 3.1**

We say that *X is (conditionally) independent of Y given* $(Z,\sigma )$ and write $X \, \,\perp \!\!\!\perp \, Y \mid (Z,\sigma )$, if for any bounded real measurable function $h(X)$, there exists a function $w(\sigma ,Z)$, measurable in $Z$, such that, for all $s\in \mathcal{S}$,

$$\begin{aligned} \mathbb {E}\{h(X)\mid Y,Z\,;\,s\}=w(s,Z) \quad \hbox {a.s.}\;[{P_{s}}]. \end{aligned}$$

##### **Definition 3.2**

We say that *X is (conditionally) independent of* $(Y,\sigma )$ *given* $Z$, and write $X \, \,\perp \!\!\!\perp \, (Y,\sigma ) \mid Z$, if for any bounded real measurable function $h(X)$, there exists a measurable function $w(Z)$ such that, for all $s \in \mathcal{S}$,

7 $$\begin{aligned} \mathbb {E}\{h(X)\mid Y, Z\,;\,s\}=w(Z) \quad \hbox {a.s.}\;[{P_s}]. \end{aligned}$$

##### *Remark 3.1*

1. Note the similarity of (7) to (6). In particular the function $w(Z)$ must not depend on the regime $s\in \mathcal{S}$ operating.
2. When $X, Y$ and $Z$ are discrete random variables, $X \, \,\perp \!\!\!\perp \, (Y,\sigma ) \mid Z$ if and only if there exists a function $w(X,Z)$ such that, for any $s \in \mathcal{S}$,
  $$\begin{aligned} P(X=x\,|\,Y=y, Z=z\,;\,s)=w(x,z) \end{aligned}$$
  whenever $$P(Y=y, Z=z\,;\,s)>0$$.
3. For each $s\in \mathcal{S}$, the equality in (7) is permitted to fail on a set $A_s$, which may vary with $s$, that has probability $0$ under $P_s$.
4. The requirement of (7) is that there exist a single function $w(Z)$ that can serve as the conditional expectation of $h(X)$ given $(Y,Z)$ in every distribution $P_s$; but this does not imply that any version of this conditional expectation under one value of $s$ will serve for all values of $s$: see Counter-example A.1 in the Appendix for a counter-example, and Dawid [4] for cases where a lack of understanding of similar problems associated with null events has led to serious errors. However we can sometimes escape this problem by imposing an additional positivity condition—see Sect. 4.1 below.

#### Connexions

In this section we impose the additional condition that the set $\mathcal{S}$ of possible regimes be finite or countable, and endow it with the $\sigma$-field $\mathcal{F}$ of all its subsets.

We can construct the product measure space $(\varOmega ^*,\mathcal{A}^*) := (\varOmega \times \mathcal{S}, \mathcal{A}\otimes \mathcal{F})$, and regard all the stochastic variables $X, Y, Z, \ldots$ as defined on $(\varOmega ^*,\mathcal{A}^*)$; moreover $\sigma$ can also be considered as a random variable on $(\varOmega ^*,\mathcal{A}^*)$.

Let $\Pi$ be a probability measure on $\mathcal{S}$, arbitrary subject only to giving positive probability $\pi (s)>0$ to each point $s\in \mathcal{S}$; and define, for any $A^* \in \mathcal{A}^*$:

8 $$\begin{aligned} P^*(A^*) = \sum _{s\in \mathcal{S}} \pi (s) P_s(A_s) \end{aligned}$$

where $A_s\! =\!\{\omega \in \varOmega : (\omega ,s)\!\in \! A^*\}$. Under $P^*$ the marginal distribution of $\sigma$ is $\Pi$, while the conditional distribution over $\varOmega$, given $\sigma \!=\! s$, is $P_s$. It is then not hard to show [1] [Theorem 3.2.5] that $X \, \,\perp \!\!\!\perp \, Y \mid (Z,\sigma )$ holds in the extended sense of Definition 3.1 if and only if the purely stochastic interpretation of the same expression holds under $P^*$; and similarly for Definition 3.2. It follows that, for the interpretations of extended conditional independence given in Sect. 3.1.2, we can continue to apply all the properties P1–P5 of Theorem 3.1. Any argument so constructed, in which all the premisses and conclusions are so interpretable, will be valid—even when some of the intermediate steps are not so interpretable (e.g., they could have the form $\sigma \, \,\perp \!\!\!\perp \, X \mid Y$).

For the purposes of this paper we will only ever need to compare two regimes at a time: the observational regime $o$ and one particular interventional regime $s$ of interest. Then the properties P1–P5 of conditional independence can always be applied, and equip us with a powerful machinery to pursue identification of interventional quantities from observational data.

#### Graphical Representations

Graphical models in the form of *influence diagrams* (IDs) can sometimes be used to represent collections of conditional independence properties among the variables (both stochastic and non-stochastic) in a problem [2, 8, 10]. We can then use graphical techniques (in particular, the *d-separation*, or the equivalent *moralization*, criterion) to derive, in a visual and transparent way, implied (extended) conditional independence properties that follow from our assumptions. We emphasize that the arrows in such an ID represent causality only indirectly, through these implied conditional independence properties, and are not otherwise to be interpreted as carrying causal meaning. In any case, a graphical representation is not always possible and never essential: all that can be achieved through the graph-theoretic properties of IDs, and more, can be achieved using the calculus of conditional independence (properties P1–P5).

## Simple Stability

We now use CI to express and explore some conditions that will allow us to perform $G$-recursion for the strategy of interest on the basis of observational data.

Consider first the conditional distribution (i) of $A_i \mid \overline{L}_{i}, \overline{A}_{i-1}\,;\, s$ as needed for (4). This term requires knowledge of the mechanism that allocates the treatment at stage $i$ in the light of the preceding variables in the information base. We assume that, for an interventional regime $s \in \mathcal{S}^*$, this distribution (degenerate for a non-randomized strategy) will be known *a priori* to the analyst, as it will be encoded in the strategy. In such a case we call $s \in \mathcal{S}^*$ a *control strategy* (with respect to the information base $\mathcal{I} = (L_1, A_1,\ldots , L_N, A_N, Y)$).

Next we consider how we might gain knowledge of the conditional distribution (ii) of $L_i \mid \overline{L}_{i-1}, \overline{A}_{i-1}\,;\, s$, as required for (5). This distribution is unknown, and we need to explore conditions that will enable us to identify it from observational data. As different distributions for the random variables in the information base apply in the different regimes, the distribution of $L_i$ given $(\overline{L}_{i-1}, \overline{A}_{i-1})$ will typically depend on the regime operating.

### **Definition 4.1**

We say that the problem exhibits *simple stability* 1 with respect to the information base $\mathcal{I} = (L_1,A_1, \ldots , L_n,A_n, Y)$ if, for each $s\in \mathcal{S}^*$, with $\sigma$ denoting the non-random regime indicator taking values in $\{o,s\}$:

9 $$\begin{aligned} L_i \, \,\perp \!\!\!\perp \, \sigma \mid (\overline{L}_{i-1}, \overline{A}_{i-1}) \quad (i =1, \ldots , n+1). \end{aligned}$$

Formally, simple stability requires that, for any bounded measurable function $f(L_i)$, there exist a single random variable $W = w(\overline{L}_{i-1}, \overline{A}_{i-1})$ that serves as a version of each of the conditional expectations $\mathbb {E}\{f(L_i)\mid (\overline{L}_{i-1}, \overline{A}_{i-1})\,;\,o\}$ and $\mathbb {E}\{f(L_i)\mid (\overline{L}_{i-1}, \overline{A}_{i-1})\,;\,s\}$. This property then extends to conditional expectations of functions of the form $f(\overline{L}_i, \overline{A}_{i-1})$. In particular, this apparently^2^ supports identification of the right-hand side of (5) with its observational counterpart, so allowing observational estimation of this expression.

Simple stability is a very strong assumption, and will be tenable only in very special cases. It will be satisfied if, in the observational regime, the action variables are physically sequentially randomized: then all unobserved potential confounding factors will, on average, be balanced between the treatment groups. Alternatively, we might accept simple stability if, in the observational regime, the allocation of treatment is decided taking into account only the domain variables in the information base and nothing more: for example, if we are observing a doctor whose treatment decisions are based only on the domain variables we are recording, and no additional unrecorded information.

**Fig. 1:**
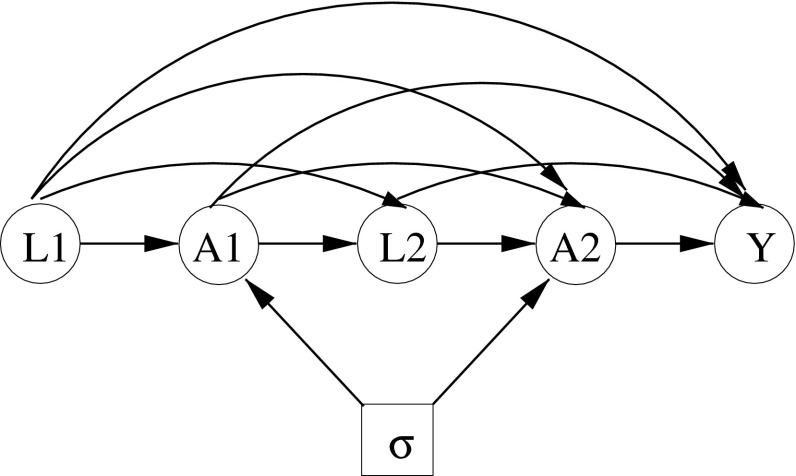
Stability

An ID describing simple stability (9) for $i = 1, 2, 3$ is shown in Fig. 1. The specific property (9) is represented by the absence of arrows from $\sigma$ to $L_1, L_2$, and $L_3 \equiv Y$.

### Positivity

We have indicated that simple stability might allow us to identify the consequence of a control strategy $s$ on the basis of data from the observational regime $o$. However, while this condition ensures the existence of a common version of the relevant conditional expectation, valid for both regimes, deriving this function from the observational regime alone might be problematic, because versions of the same conditional expectation can differ on events of probability 0, and we have not ruled out that an event having probability 0 in one regime might have positive probability in another. Thus we can only obtain the desired function from the observational regime on a set that has probability 1 in the observational regime; and this might not have probability 1 in the interventional regime—see Counter-example A.1 in the Appendix for a simple example of this.

To evade this problem, we can impose a condition requiring an event to have zero probability in the interventional regime whenever it has zero probability in the observational regime:

#### **Definition 4.2**

We say the problem exhibits *positivity* or *absolute continuity* if, for any interventional regime $s \in \mathcal{S}^*$, the joint distribution of $(\overline{L}_n, \overline{A}_n, Y)$ under $P_s$ is absolutely continuous with respect to that under $P_o$, i.e.:

10 $$\begin{aligned} P_{s}(E) > 0 \Rightarrow P_{o}(E)> 0 \end{aligned}$$

for any event $E$ defined in terms of $(\overline{L}_n, \overline{A_n}, Y)$.

Suppose we have both simple stability and positivity, and consider a bounded function $h(L_i)$. Let $W = w(\overline{L}_{i-1}, \overline{A}_{i-1})$ be any variable that serves both as a version of $\mathbb {E}\{h(L_i)\mid \overline{L}_{i-1}, \overline{A}_{i-1}\,;\,o\}$ and as a version of $\mathbb {E}\{h(L_i)\mid \overline{L}_{i-1}, \overline{A}_{i-1}\,;\,s\}$; such a variable is guaranteed to exist by (9). Let $V = v(\overline{L}_{i-1}, \overline{A}_{i-1})$ be any version of $\mathbb {E}\{h(L_i)\mid \overline{L}_{i-1}, \overline{A}_{i-1}\,;\,o\}$. Since $W$ too is a version of $\mathbb {E}\{h(L_i)\mid \overline{L}_{i-1}, \overline{A}_{i-1}\,;\,o\}$, $V=W, \hbox {a.s.}\;[{P_o}]$. Hence, by (10), $V=W, \hbox {a.s.}\;[{P_s}]$. But since $W$ is a version of $\mathbb {E}\{h(L_i)\mid \overline{L}_{i-1}, \overline{A}_{i-1}\,;\,s\}$, so too must be $V$. So we have shown that any version of a conditional expectation calculated under $P_o$ will also serve this purpose under $P_s$. In particular, when effecting the $G$-computation algorithm of Sect. 2.3.1, in (5) we are fully justified in replacing the conditional expectation under $P_s$ by (any version of) its counterpart under $P_o$—which we can in principle estimate from observational data.

#### Difficulties with Continuous Actions

When all variables are discrete, positivity will hold if and only if every partial history that can occur with positive probability in the interventional regime also has a positive probability in the observational regime. In particular, this will hold for every interventional regime if every possible partial history can occur with positive probability in the observational regime.

Even in this case we might well need vast quantities of observational data to get good estimates of all the probabilities needed for substitution into the $G$-recursion algorithm—that is the reason for our qualification “in principle” at the end of Sect. 4.1. In practice, even under positivity we would generally need to impose some smoothness or modelling assumptions to get reasonable estimates of the required observational distributions. However we do not explore these issues here, merely noting that, given enough data to estimate these observational distributions, positivity allows us to transfer them to the interventional regime.

When however we are dealing with continuous action variables—as, for example, the dose of a medication—the positivity condition may become totally unreasonable. For a very simple example, consider a single continuous action variable $A$ and response variable $Y$. We might want to transfer the conditional expectation $\mathbb {E}(Y\,|\,A)$ from the observational regime $o$, in which $A$ arises from a continuous distribution, to an interventional regime $s$, in which it is set to a fixed value, $A=a_0$. However, if we take any version of $\mathbb {E}(Y\,|\,A;o)$ and change it, to anything we want, at the single point $A=a_0$, we will still have a version of $\mathbb {E}(Y\,|\,A;o)$. So we are unable to identify the desired $\mathbb {E}(Y\,|\,A;s)$ This is due to the failure of positivity, since the 1-point interventional distribution of $A$ is not absolutely continuous with respect to the continuous observational distribution of $A$. Positivity here would require that there be a positive probability of observing the exact value $a_0$ in the observational regime. But it would not generally be reasonable to impose such a condition, and quite impossible to do so for every value $a_0$, that we might be potentially interested in setting for $A$.

In such a case we might make progress by imposing further structure, such as a model for $\mathbb {E}(Y\,|\,A;o)$ that is a continuous function of $A$, so identifying a preferred version of this. Here however we shall avoid such problems by only considering problems in which all action variables are discrete. Then we shall have positivity whenever every action sequence $\overline{a}$ having positive interventional probability also has positive observational probability, and the (uniquely defined) conditional interventional distribution of all the non-action variables, given $\overline{A} = \overline{a}$, is absolutely continuous with respect to its observational counterpart. This will typically not be an unreasonable requirement. We note that this set-up is still more general than usual formulations of $G$-recursion, which explicitly or implicitly assume that all variables are discrete.

## Sequential Ignorability

As we have alluded, simple stability will often not be a compelling assumption, for example because of the suspected presence of unmeasured confounding variables, and we might not be willing to accept it without further justification. Here we consider conditions that might seem more acceptable, and investigate when these will, after all, imply simple stability—thus supporting the application of $G$-recursion.

### Extended Stability and Extended Positivity

Let $\mathcal {U}$ denote a set of variables that, while they might potentially influence actions taken under the observational regime, are not available to the decision maker, and so are not included in his information base $\mathcal{I}:= (L_1, A_1,\ldots , L_n, A_n, L_{n+1}\equiv Y)$. We define the *extended information base* $\mathcal{I}':= (L_1, U_1, A_1,\ldots , L_n, U_n, A_n, L_{n+1})$, with $U_i$ denoting the variables in $\mathcal{U}$ realized just before action $A_i$ is taken. However, while thus allowing $U_i$ to influence $A_i$ in the observational regime, we still only consider interventional strategies where there is no such influence—since the decision maker does not have access to the $(U_i)$. This motivates an extended formal definition of “control strategy” in this context:

#### **Definition 5.1 (Control strategy)**

A regime $s$ is a *control strategy* if

11 $$\begin{aligned} A_i \, \,\perp \!\!\!\perp \, \overline{U}_i \mid (\overline{L}_i, \overline{A}_{i-1}\,;\, s) \quad (i = 1,\ldots , n) \end{aligned}$$

and in addition, the conditional distribution of $A_i$, given $(\overline{L}_{i}, \overline{A}_{i-1})$, under regime $s$, is known to the analyst.

We again denote the set of interventional regimes corresponding to the control strategies under consideration by $\mathcal{S}^*$.

#### **Definition 5.2**

We say that the problem exhibits *extended stability* (with respect to the extended information base $\mathcal{I}'$) if, for any $s\in \mathcal{S}^*$, with $\sigma$ denoting the non-random regime indicator taking values in $\{o,s\}$:

12 $$\begin{aligned} (L_i,U_i) \, \,\perp \!\!\!\perp \, \sigma \mid ({\overline{L}_{i-1}, \overline{U}_{i-1}, \overline{A}_{i-1}}) \quad (i =1, \ldots , n+1). \end{aligned}$$

**Fig. 2:**
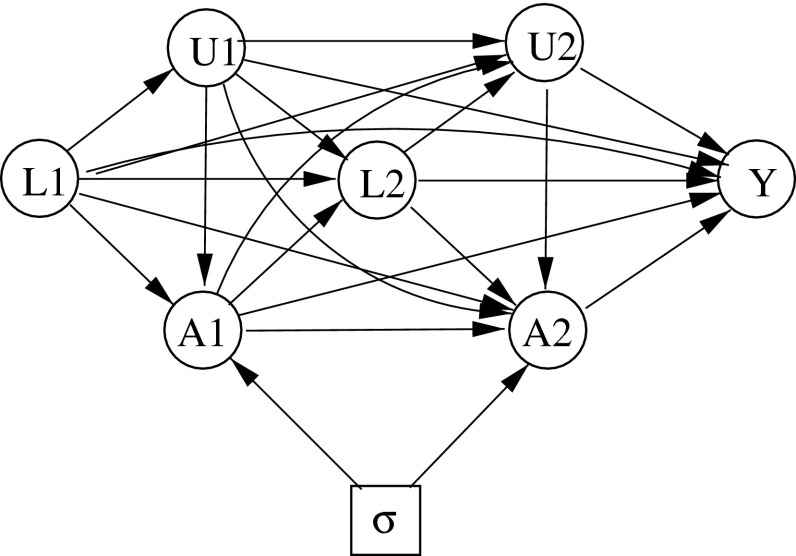
Extended stability

Extended stability is formally the same as simple stability, but using a different information base, where $L_i$ is expanded to $(L_i, U_i)$. The real difference is that the extended information base is not available to the decision maker in the interventional regime, so that his decisions can not take account of the $(U_i)$. An ID faithfully representing property (12) for $i = 1, 2, 3$ is shown in Fig. 2 3. The property (12) is represented by the absence of arrows from $\sigma$ to $L_1, U_1, L_2, U_2$ and $Y$. However, the diagram does not explicitly represent the additional property (11), which implies that, *when* $\sigma = s$, the arrows into $A_1$ from $U_1$ and into $A_2$ from $U_1$ and $U_2$ can be dropped.

To evade problems with events of zero probability, we can extend Definition 4.2:

#### **Definition 5.3**

We say the problem exhibits *extended positivity* if, for any $s \in \mathcal{S}^*$, the joint distribution of $(\overline{U}_n, \overline{L}_n, \overline{A}_n, Y)$ under $P_s$ is absolutely continuous with respect to that under $P_o$, i.e.

13 $$\begin{aligned} P_{s}(E) > 0 \Rightarrow P_{o}(E)> 0 \end{aligned}$$

for any event $E$ defined in terms of $(\overline{L}_n, \overline{U}_n, \overline{A}_n, Y)$.

### Sequential Randomization

Extended stability represents the belief that, for each $i$, the conditional distribution of $(L_i,U_{i})$, given all the earlier variables $(\overline{L}_{i-1}, \overline{U}_{i-1}, \overline{A}_{i-1})$ in the extended information base, is the same in the observational regime as in the interventional regime. This will typically be defensible if we can argue that we have included in $\mathcal{L}\cup \mathcal{U}$ all the variables influencing the actions in the observational regime.

However extended stability, while generally more defensible than simple stability, typically does not imply simple stability, which is what is required to support $G$-recursion. But it may do so if we impose additional conditions. Here and in Sect. 5.3 below we explore two such conditions.

Our first is the following:

#### **Condition 5.3 (Sequential randomization)**

14 $$\begin{aligned} A_i \, \,\perp \!\!\!\perp \, \overline{U}_i \mid (\overline{L}_i, \overline{A}_{i-1}\,;\, o) \quad (i = 1, \ldots , n). \end{aligned}$$

Taking account of (11), we see that (14) is equivalent to:

15 $$\begin{aligned} A_i \, \,\perp \!\!\!\perp \, \overline{U}_i \mid (\overline{L}_i, \overline{A}_{i-1}\,;\, \sigma ) \quad (i = 1,\ldots , n) \end{aligned}$$

where $\sigma$ takes values in $\mathcal{S} = \{o\} \cup \mathcal{S}^*$.

Under sequential randomization, the observational distribution of $A_i$, given the earlier variables in the information base, would be unaffected by further conditioning on the earlier unobservable variables, ${\overline{U}_i}$. Hence the $(U_i)$ are redundant for explaining the way in which actions are determined in the observational regime. While this condition will hold under a control strategy, in the observational regime it requires that the only information that has been used to assign the treatment at each stage is that supplied by the observable variables. For example, sequential randomization will hold if the actions are physically sequentially randomized within all levels of the earlier variables in the information base. The following result is therefore unsurprising.

#### **Theorem 5.1**

Suppose we have both extended stability, (12) and sequential randomization, (15). Then we have simple stability, (9).

**Fig. 3:**
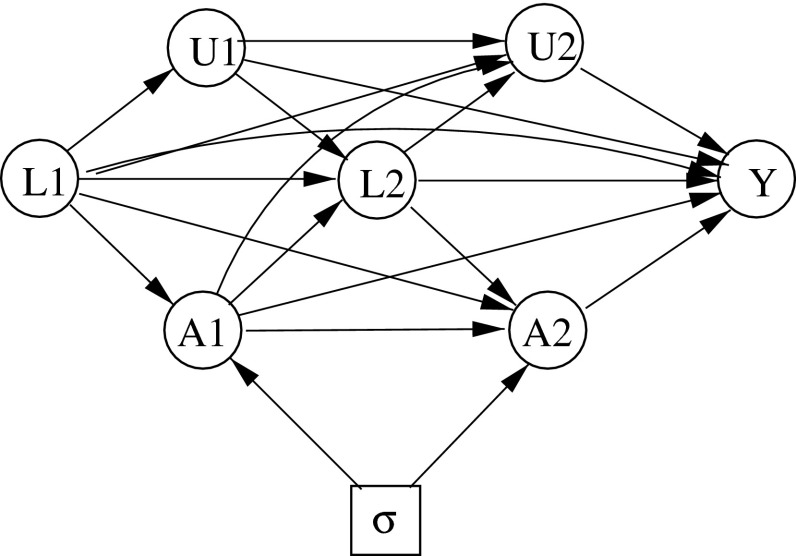
Sequential randomization

An ID faithfully representing the conditional independence relationships assumed in Theorem 5.1, for $i=1,2,3$, is shown in Fig. 3. Figure 3 can be obtained from Fig. 2 on deleting the arrows into $A_1$ from $U_1$ and into $A_2$ from $U_1$ and $U_2$, so representing (15). (However, as we shall see below in Sect. 5.3, in general such “surgery” on IDs can be hazardous.)

The conditional independence properties (9) characterizing simple stability can now be read off from Fig. 3, by applying the $d$-separation or moralization criteria. For a formal algebraic proof of Theorem 5.1, using just the axioms of conditional independence as given in Theorem 3.1, see Theorem 6.1 of Dawid and Didelez [9]^4^.

#### **Corollary 5.1**

Suppose we have extended stability, sequential randomization, and simple positivity. Then we can apply $G$-recursion to compute the consequence of a strategy $s\in \mathcal{S}^*$.

### Sequential Irrelevance

Consider now the following alternative condition:

#### **Condition 5.4 (Sequential Irrelevance)**

16 $$\begin{aligned} L_i \, \,\perp \!\!\!\perp \, \overline{U}_{i-1} \mid (\overline{L}_{i-1}, \overline{A}_{i-1}\,;\, \sigma ) \quad (i = 1, \ldots , n+1). \end{aligned}$$

Under sequential irrelevance, in both regimes the conditional distribution of the observable variable(s) at stage $i$ is unaffected by the history of unobservable variables up to the previous stage $i-1$, given the domain variables in the information base up to the previous stage. In contrast to (15), (16) permits the unobserved variables that appear in earlier stages to influence the next action $A_i$ (which can only happen in the observational regime)—but not the development of the subsequent observable variables (including the ultimate response variable $Y$). This will hold when at each stage $i$ the unobserved variable $U_i$ does not affect the development of future $L$’s: for example, $U_i$ might represent the inclination of the patient to take the current treatment $A_i$. In general, the validity of this assumption will have to be justified in the context of the problem under study.

**Fig. 4:**
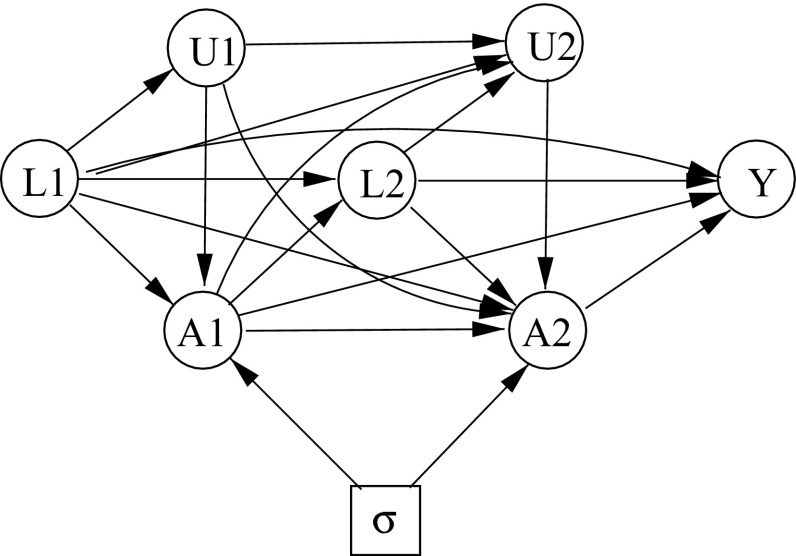
Sequential irrelevance?

By analogy with the passage from Figs. 2 to 3, we might attempt to represent the additional assumption (16) by removing from Fig. 2 all arrows from $U_j$ to $L_i$ ($j<i$). This would yield Fig. 4. On applying $d$-separation or moralization to Fig. 4 we could then deduce the simple stability property (9). However, this approach is not valid, since Fig. 4 encodes the property $L_2 \, \,\perp \!\!\!\perp \, \sigma \mid (L_1, A_1)$, which can not be derived from (12) and (16) using only the “axioms” of Theorem 3.1. In fact there is no ID that faithfully represents the combination of the properties (12) and (16), since these do not form a recursive system [9] [Sect. 7.1]. And indeed, in full generality, simple stability is not implied by extended stability, (12), together with sequential irrelevance, (16), as the following counter-example demonstrates.

#### **Counter-example 5.5**

Take $n=1, \mathcal{L} = \emptyset$ and $\mathcal{U} = \{U\}$. The extended information base is $\mathcal{I}'=(U, A, Y)$. We suppose that, in both the observational regime $o$ and the interventional regime $s, Y = 1$ if $A=U$, else $Y=0$. Also, in each regime, the marginal distribution of $U$ is uniform on [0,1]. It remains to specify the distribution of $A$, given $U$: we assume that, in regime $o$, $A\equiv U$, while in regime $s, A$ is uniform on [0,1], independently of $U$.

**Fig. 5:**
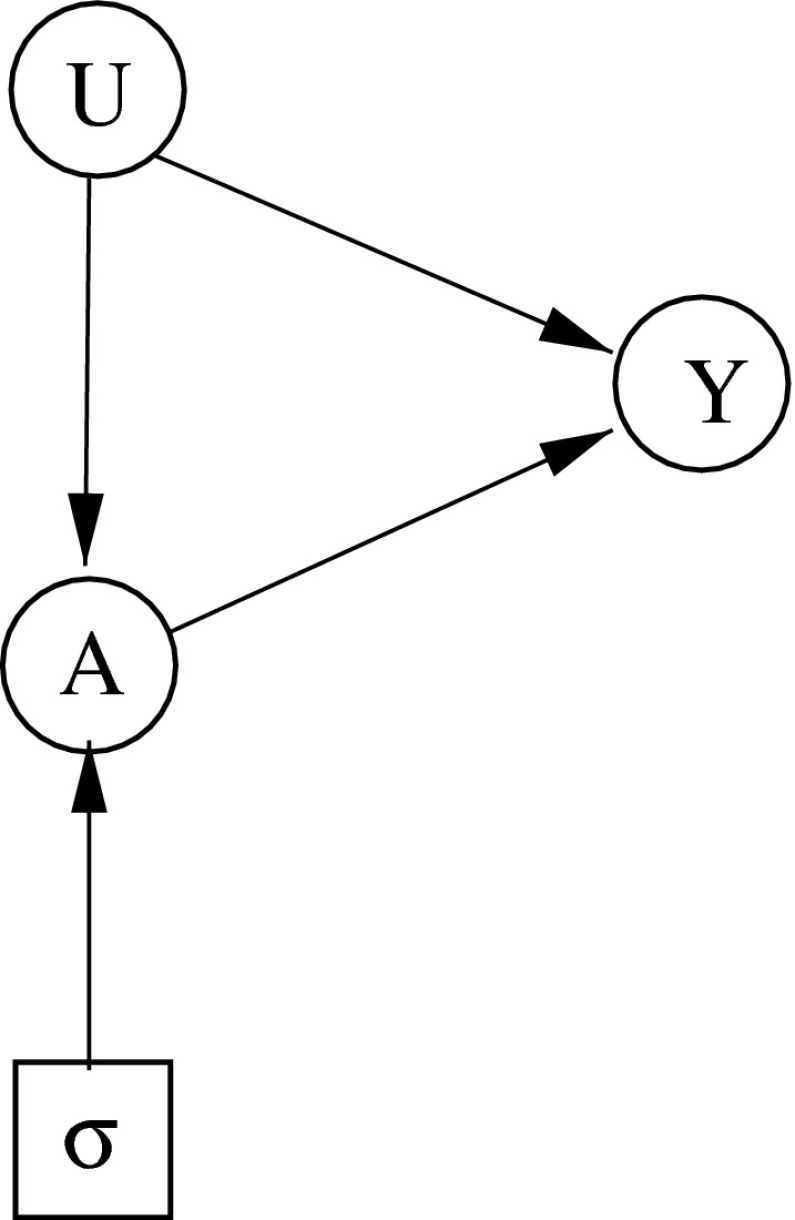
Counter-example

It is readily seen that $U \,\,\perp \!\!\!\perp \, \sigma$ and $Y \, \,\perp \!\!\!\perp \, \sigma \mid (U,A)$. Thus we have extended stability, (12), as represented by the ID of Fig. 5.

Also, since $U \,\,\perp \!\!\!\perp \, A$ in regime $s$, (11) holds, so $s$ is a control strategy. Finally, in regime $o, Y=1$ a.s., while in regime $s$, $Y=0$ a.s. Because these are both degenerate distributions, trivially $Y \, \,\perp \!\!\!\perp \, U \mid (A,\sigma )$, and we have sequential irrelevance. However, because they are different distributions, $Y \not \!\!\,\perp \!\!\!\perp _{}\, \sigma \mid A$: so we do *not* have simple stability, (9). In particular, we can not remove the arrow from $U$ to $Y$ in Fig. 5, since this would encode the false property $Y \, \,\perp \!\!\!\perp \, \sigma \mid A$. $\square$

So, if we wish to deduce simple stability from extended stability and sequential irrelevance, further conditions, and a different approach, will be required.

In Theorem 6.2 of Dawid and Didelez [9] it is shown that this result does follow if we additionally impose the extended positivity condition of Definition 5.3; and then we need only require sequential irrelevance, (16), to hold for the observational regime $\sigma = o$.

However, in Sect. 6 below we show that, if we restrict attention to discrete variables, no further conditions are required for the result to hold. And in this case we need only require sequential irrelevance to hold for the interventional regime $\sigma = s$.

## Discrete Case

In this section we assume all variables are discrete, and denote $P(\overline{A}=\overline{a}, \overline{L} = \overline{l})$ by $p(\overline{a}, \overline{l})$, etc.

To control null events, we need the following lemma:

### **Lemma 6.1**

Let all variables be discrete. Suppose that we have extended stability, (12), and let $s$ be a control strategy, so that (11) holds. Then, for any $(\overline{u}_k, \overline{l}_k,\overline{a}_k)$ such that

$$\mathbf{A}_\mathbf{k}$$: $$p(\overline{l}_k,\overline{a}_k\,;\,s)>0$$, and

$$\mathbf{B} _\mathbf{k}$$: $$p(\overline{u}_k,\overline{l}_k,\overline{a}_k\,;\,o) > 0 $$, we have

$$\mathbf{C} _ \mathbf{k}$$: $$p(\overline{u}_k,\overline{l}_k,\overline{a}_k\,;\,s)>0$$.

### *Proof*

Let $H_{k}$ denote the assertion that $\mathbf{A_k}$ and $\mathbf{B_k}$ imply $\mathbf{C_k}$. We establish $H_k$ by induction.

To start, we note that $H_0$ holds vacuously.

Now suppose $H_{k-1}$ holds. Assume further $\mathbf{A_k}$ and $\mathbf{B_k}$. Together these conditions imply that all terms appearing throughout the following argument are positive.

We have

17 $$\begin{aligned} p(\overline{u}_{k}, \overline{l}_{k}, \overline{a}_{k}\,;\,s)&= p(\overline{u}_{k}\mid \overline{l}_{k},\overline{a}_{k}\,;\,s)\,p(\overline{l}_{k},\overline{a}_{k}\,;\,s)\nonumber \\&= p(\overline{u}_{k}\mid \overline{l}_{k},\overline{a}_{k-1}\,;\,s)\, p(\overline{l}_{k},\overline{a}_{k}\,;\,s) \\&= \frac{p(\overline{u}_{k},\overline{l}_{k},\overline{a}_{k-1}\,;\,s)}{p(\overline{l}_{k},\overline{a}_{k-1}\,;\,s)} p(\overline{l}_{k},\overline{a}_{k}\,;\,s) \nonumber \\&= p(u_{k},l_{k}\mid \overline{u}_{k-1},\overline{l}_{k-1},\overline{a}_{k-1}\,;\,s) \nonumber \\&\quad \times \,\frac{p(\overline{u}_{k-1},\overline{l}_{k-1},\overline{a}_{k-1}\,;\,s)\,p(\overline{l}_{k},\overline{a}_{k}\,;\,s)}{p(\overline{l}_{k},\overline{a}_{k-1}\,;\,s)}\nonumber \\&= p(u_{k},l_{k}\mid \overline{u}_{k-1},\overline{l}_{k-1},\overline{a}_{k-1}\,;\,o) \nonumber \\&\quad \times \,\frac{p(\overline{u}_{k-1},\overline{l}_{k-1},\overline{a}_{k-1}\,;\,s)\,p(\overline{l}_{k},\overline{a}_{k}\,;\,s)}{p(\overline{l}_{k},\overline{a}_{k-1}\,;\,s)} \end{aligned}$$

18 $$\begin{aligned}&= \frac{p(\overline{u}_{k},\overline{l}_{k},\overline{a}_{k-1}\,;\,o)}{p(\overline{u}_{k-1},\overline{l}_{k-1},\overline{a}_{k-1}\,;\,o)} \nonumber \\&\quad \times \,\frac{p(\overline{u}_{k-1},\overline{l}_{k-1},\overline{a}_{k-1}\,;\,s)\,p(\overline{l}_{k},\overline{a}_{k}\,;\,s)}{p(\overline{l}_{k},\overline{a}_{k-1}\,;\,s)}\nonumber \\&> 0. \nonumber \end{aligned}$$

Here (17) holds by (11) and (18) holds by (12). The induction is established. $\square$

### **Theorem 6.1**

Suppose the conditions of Lemma 6.1 apply, and, further, that we have sequential irrelevance in the interventional regime $s$:

19 $$\begin{aligned} L_i \, \,\perp \!\!\!\perp \, \overline{U}_{i-1} \mid (\overline{L}_{i-1}, \overline{A}_{i-1}\,;\, s) \quad (i = 1, \ldots , n+1). \end{aligned}$$

Then the simple stability property (9) holds.

### *Proof*

The result will be established if we can show that, for any $l_{i}$, we can find a function $w(\overline{L}_{i-1}, \overline{A}_{i-1})$ such that, for both $\sigma = o$ and $\sigma = s$,

$$\begin{aligned} p(l_{i} \mid \overline{l}_{i-1},\overline{a}_{i-1}\,;\,\sigma )=w(\overline{l}_{i-1},\overline{a}_{i-1}) \end{aligned}$$

whenever $$p(\overline{l}_{i-1},\overline{a}_{i-1}\,;\,\sigma )>0$$.

This is trivially possible if either regime gives probability 0 to $(\overline{l}_{i-1},\overline{a}_{i-1})$. So suppose $p(\overline{l}_{i-1},\overline{a}_{i-1}\,;\,\sigma )>0$ for both regimes. Then

20 $$\begin{aligned} p(l_{i} \mid \overline{l}_{i-1},\overline{a}_{i-1}\,;\,o) =\sum _{\overline{u}_{i-1}}{}^{'}p(l_{i} \mid \overline{u}_{i-1}, \overline{l}_{i-1},\overline{a}_{i-1}\,;\,o)\, \times \, p(\overline{u}_{i-1} \mid \overline{l}_{i-1},\overline{a}_{i-1}\,;\,o)\nonumber \\ \end{aligned}$$

where $\sum '$ denotes summation restricted to terms for which $p(\overline{u}_{i-1},\overline{l}_{i-1},\overline{a}_{i-1}\,;\,o) > 0$—and so, by Lemma 6.1, $p(\overline{u}_{i-1},\overline{l}_{i-1},\overline{a}_{i-1}\,;\,s)>0$. Then by (12),

21 $$\begin{aligned} p(l_{i} \mid \overline{l}_{i-1},\overline{a}_{i-1}\,;\,o)&= \sum _{\overline{u}_{i-1}}{}^{'}p(l_{i} \mid \overline{u}_{i-1},\overline{l}_{i-1},\overline{a}_{i-1}\,;\,s)\, \times \,p(\overline{u}_{i-1} \mid \overline{l}_{i-1},\overline{a}_{i-1}\,;\,o) \nonumber \\&= \sum _{\overline{u}_{i-1}}{}^{'}p(l_{i} \mid \overline{l}_{i-1},\overline{a}_{i-1}\,;\,s)\times p(\overline{u}_{i-1} \mid \overline{l}_{i-1},\overline{a}_{i-1}\,;\,o) \\&= p(l_{i} \mid \overline{l}_{i-1},\overline{a}_{i-1}\,;\,s) \nonumber \end{aligned}$$

where (21) holds by (19). Thus we can take

$$\begin{aligned} w(\overline{l}_{i-1},\overline{a}_{i-1}):=p({l}_{i} \mid \overline{l}_{i-1},\overline{a}_{i-1}\,;\,s) \end{aligned}$$

to conclude the proof. $\square$

Counter-example A.2 in the Appendix demonstrates that, even in this discrete case, to deduce simple stability under the conditions of Lemma 6.1 it is not sufficient to impose sequential irrelevance only for the observational regime $o$.

We summarise our findings on sequential irrelevance in the following corollary:

### **Corollary 6.1**

Suppose we have extended stability, sequential irrelevance, and extended positivity. Then we can apply $G$-recursion to compute the consequence of a strategy $s\in \mathcal{S}^*$. In the special case that all variables in the extended information base are discrete, we can replace the condition of extended positivity by simple positivity.

## Conclusion

The decision-theoretic approach to causal inference focuses on the possibilities for transferring probabilistic information between different stochastic regimes. In this paper we have developed a formal underpinning for this approach, based on an extension of the axiomatic theory of conditional independence to include non-stochastic variables. This formal foundation now supplies a rigorous justification for various more informal arguments that have previously been presented [3, 8, 9].

By applying this theory to the problem of dynamic treatment assignment, we have shown how, and under what additional conditions, the assumptions of sequential randomization or sequential irrelevance can support observational identification of the consequence of some treatment strategy under consideration. Specifically, in order to identify the consequence of a control strategy directly from observational data by means of $G$-recursion, we should like to establish the properties of simple positivity and simple stability. Simple positivity will often be a reasonable assumption to impose directly, at any rate when all the action variables are discrete. However, simple stability may be harder to justify. Instead, we might begin with the weaker and more readily justifiable assumption of extended stability. We have investigated when, in combination with appropriate additional conditions, extended stability will imply simple stability.

Our first additional condition is sequential randomization. Extended stability and sequential randomization together imply simple stability, even without imposing any positivity assumption. (However, for the purposes of complete identification of a control strategy from observational data using $G$-recursion, we still need to require simple positivity, in order to guarantee that any version of the desired conditional expectation that can be recovered from the observational regime can simultaneously serve as a version for the interventional regime.)

The second condition studied is sequential irrelevance. However, extended stability together with sequential irrelevance are not in general sufficient to imply simple stability, and a further assumption of extended positivity is typically also needed. Since extended positivity implies simple positivity, these conditions are jointly sufficient to enable identification of a control strategy from observational data using $G$-recursion. However, since the property of extended positivity involves unobservable variables, justifying this assumption can be problematic. We have shown that, in the special case that all the random variables involved are discrete, we can dispense with this additional assumption. (Of course, we will still need the weaker assumption of simple positivity to support $G$-recursion.) In the presence of continuous random variables, we have shown, by means of a counterexample, that the assumption of extended positivity may be indispensible.

In the light of our analysis, we offer the following advice to the analyst who wishes to use observational data in order to evaluate a control strategy: Examine carefully which of the assumptions enabling application of $G$-recursion can be sensibly justified in the context of the problem under study. In particular, can simple stability reasonably be assumed? — since otherwise (as we discussed in Sect. 2.2) a naïve analysis may suffer from bias.

Whereas for data obtained from a randomized control trial the assumption of simple stability may be robustly defensible, for more typical observational regimes the analyst would need to be able to present a good argument for assuming simple stability. Our conditions of sequential randomization and sequential irrelevance, together with the additional supporting conditions we have identified, supply a possible route to making such an argument.

## Appendix: The Need for Positivity

### **Counter-example A.1**

The following counter-example illustrates what can go wrong when we do not have positivity: even when a property such as (7) holds, we can not use just any version of the conditional expectation in one regime to serve as a version of this conditional expectation in another regime.

Consider a sequential decision problem of $n=2$ stages with domain variables $L_1, A$ and $L_2$, where $A$ is a binary variable with $A=0$ denoting no treatment and $A=1$ denoting treatment. In the observational regime $o$, the treatment is never given: $P_{o}(A=0)=1$; while in the interventional regime $s$, the treatment is always given: $P_{s}(A=1)=1$. We thus have failure of the positivity requirement of Definition 4.2.

Suppose that,in both regimes, $L_1= 0$ or $1$ each with probability $1/2$, and $L_2 = L_1 + A$. Then, with $\sigma$ denoting the regime indicator taking values in $\mathcal{S}= \{o, s\}$, we trivially have $L_{2} \, \,\perp \!\!\!\perp \, \sigma \mid (L_{1}, A)$.

Now consider the variables

$$\begin{aligned} W_{o} = \left\{ \begin{array}{l@{\quad }l} L_1 &{} \text {if } A=0 \\ 0 &{} \text {if } A=1 \end{array} \right. \end{aligned}$$

and

$$\begin{aligned} W_{s} = \left\{ \begin{array}{l@{\quad }l} 2 &{} \text {if } A=0 \\ L_1+1 &{} \text {if } A=1. \end{array} \right. \end{aligned}$$

Then $W_{o}=L_2 \text { a.s. }[{P_o}]$, so $W_o$ serves as a version of $\mathbb {E}(L_2\,|\,L_1,A\,;\,o)$; also $W_{s}=L_2 \text { a.s. }[{P_s}]$, so $W_s$ serves as a version of $\mathbb {E}(L_2\,|\,L_1,A\,;\,s)$. However, almost surely under both $P_o$ and $P_s$, $W_{o} \ne W_{s}$, and neither of these variables supplies a version of $\mathbb {E}(L_2\,|\,L_1,A)$ simultaneously valid in both regimes. $\square$

### **Counter-example A.2**

In Sect. 6 we have seen that, when all random variables are discrete and the conditions of Lemma 6.1 are satisfied, in order to be able to deduce simple stability it is sufficient to require sequential irrelevance only for the interventional regime. However, without the positivity assumption simple stability does not follow if, additionally to the requirements of Lemma 6.1, we instead require sequential irrelevance only for the observational regime.

Consider a sequential decision problem of $n=2$ stages with extended information base $\mathcal{I}':= (U_1, A_1,L_2=Y)$; $L_1$ and $U_2$ are trivial and so absent. The joint distribution of the variables in $\mathcal{I}'$ in the two regimes $\sigma = o$ or $s$ is supposed given by Table 1, where the probabilities are to be taken over 1500 $\left( \mathrm{e.g.} P(U_1=0,A_1=1,Y=0\,;\,s)= \frac{252}{1500}\right)$.

The reader may check that extended stability, (12), holds, and that $s$ is a control strategy: (11) holds. Also, sequential irrelevance, (16), holds for the observational regime, though not the interventional regime. But simple stability, (9), does not hold. $\square$

**Table 1:** Sequential irrelevance in the observational regime

	$\sigma =o$	$\sigma =s$
$P(U_1=0,A_1=0,Y=0)$	0	98
$P(U_1=0,A_1=0,Y=1)$	0	77
$P(U_1=0,A_1=1,Y=0)$	315	252
$P(U_1=0,A_1=1,Y=1)$	560	448
$P(U_1=1,A_1=0,Y=0)$	50	25
$P(U_1=1,A_1=0,Y=1)$	200	100
$P(U_1=1,A_1=1,Y=0)$	135	180
$P(U_1=1,A_1=1,Y=1)$	240	320

## Footnotes

1 This definition is slightly weaker than that of Dawid and Didelez [9], as we are only requiring a common version of the corresponding conditional expectations between each single control strategy and the observational regime. We do not require that there exist one function that can serve as common version across all regimes simultaneously.

2 but see Sect. 4.1 below.

3 Note that the IDs in this paper differ from those in Dawid and Didelez [9].

4 Note that, in either of these approaches, we can restrict $\sigma$ to the two values $o$ and $s$, so fully justifying treating the non-stochastic variable $\sigma$ as if it were stochastic.
